# Prevalence and impact of multidrug-resistant bacteria in solid cancer patients with bloodstream infection: a 25-year trend analysis

**DOI:** 10.1128/spectrum.02961-23

**Published:** 2024-08-28

**Authors:** Carlos Lopera, Patricia Monzó, Tommaso Francesco Aiello, Mariana Chumbita, Olivier Peyrony, Antonio Gallardo-Pizarro, Cristina Pitart, Guillermo Cuervo, Laura Morata, Marta Bodro, Sabina Herrera, Ana Del Río, José Antonio Martínez, Alex Soriano, Pedro Puerta-Alcalde, Carolina Garcia-Vidal

**Affiliations:** 1 Infectious Diseases Department, Hospital Clínic de Barcelona, Barcelona, Spain; 2 Emergency Department, Hôpital Saint-Louis, Assistance Publique - Hôpitaux de Paris, Paris, France; 3 Microbiology Department, Hospital Clínic de Barcelona, Barcelona, Spain; 4 Universitat de Barcelona, Barcelona, Spain; Emory University School of Medicine, Atlanta, Georgia, USA

**Keywords:** bloodstream infections, solid cancer, inappropriate empirical antibiotic therapy, Gram-negative bacilli, multidrug-resistant

## Abstract

The study aimed to describe the epidemiology of multidrug-resistant (MDR) bacteria among solid cancer (SC) patients with bloodstream infections (BSIs), evaluating inappropriate empiric antibiotic treatment (IEAT) use and mortality trends over a 25-year period. All BSI occurrences in adult SC patients at a university hospital were analyzed across five distinct five-year intervals. MDR bacteria were classified as extended-spectrum beta-lactamases (ESBL)-producing and/or Carbapenem-resistant Enterobacterales, non-fermenting Gram-negative bacilli (GNB) resistant to at least three antibiotic classes, methicillin-resistant *Staphylococcus aureus* (MRSA), and Vancomycin-resistant *Enterococci*. A multivariate regression model identified the risk factors for MDR BSI. Of 6,117 BSI episodes, Gram-negative bacilli (GNB) constituted 60.4% (3,695/6,117), being the most common are *Escherichia coli* with 26.8% (1,637/6,117), *Klebsiella* spp. with 12.4% (760/6,117), and *Pseudomonas aeruginosa* with 8.6% (525/6,117). MDR-GNB accounted for 644 episodes (84.8% of MDR or 644/759), predominantly ESBL-producing strains (71.1% or 540/759), which escalated significantly over time. IEAT was administered in 24.8% of episodes, mainly in MDR BSI, and was associated with higher mortality (22.9% vs. 14%, *P* < 0.001). Independent factors for MDR BSI were prior antibiotic use [odds ratio (OR) 2.93, confidence interval (CI) 2.34–3.67], BSI during antibiotic treatment (OR 1.46, CI 1.18–1.81), biliary (OR 1.84, CI 1.34–2.52) or urinary source (OR 1.86, CI 1.43–2.43), admission period (OR) 1.28, CI 1.18–1.38, and community-acquired infection (OR 0.57, CI 0.39–0.82). The study showed an increase in MDR-GNB among SC patients with BSI. A quarter received IEAT, which was linked to increased mortality. Improving risk assessment for MDR infections and the judicious prescription of empiric antibiotics are crucial for better outcomes.

**IMPORTANCE:**

Multidrug-resistant (MDR) bacteria pose a global public health threat as they are more challenging to treat, and they are on the rise. Solid cancer patients are often immunocompromised due to their disease and cancer treatments, making them more susceptible to infections. Understanding the changes and trends in bloodstream infections in solid cancer patients is crucial, to help physicians make informed decisions about appropriate antibiotic therapies, manage infections in this vulnerable population, and prevent infection. Solid cancer patients often require intensive and prolonged treatments, including surgery, chemotherapy, and radiation therapy. Infections can complicate these treatments, leading to treatment delays, increased healthcare costs, and poorer patient outcomes. Investigating new strategies to combat MDR infections and researching novel antibiotics in these patients is of paramount importance to avoid these negative impacts.

## INTRODUCTION

In recent decades, oncology has undergone a major revolution with the introduction of increasingly personalized treatments. Such therapies have resulted in a significant decrease in toxicities and an optimal improvement in the survival of patients with different types of solid cancer (SC) (1). However, infections remain a significant complication for these patients because of their high morbidity and mortality (2, 3).

Bloodstream infections (BSIs) are one of the most severe infectious diseases for these patients (4–7). In recent years, the rapid emergence of multidrug-resistant microorganisms (8–10) has posed a major challenge with respect to the decisions about empiric antibiotic treatments (11, 12). This holds especially true because these patients have a closer relationship with the healthcare environment. This exposure often leads to a frequent contact with multidrug-resistant (MDR) bacteria and extensive use of antibiotics that contributes to the selective pressure promoting the development of new resistant strains (8–10). Yet, knowledge on the current epidemiology of BSI in patients with SC is scarce.

In this study, we aimed to describe the changing epidemiology focusing on the microbial distribution, clinical features, and incidence of MDR bacteria in our 25-year cohort of patients with SC and BSI. Moreover, we detailed the percentage of patients who received inappropriate empiric antibiotic treatment (IEAT), as well as the 30-day mortality trend.

## MATERIALS AND METHODS

### Setting, data collection and study population

We performed this study at the Hospital Clinic in Barcelona (Spain), a 700-bed university center that provides broad and specialized medical, surgical, and intensive care for an urban population of 500,000 people. Our center annually treats more than 2,000 patients with SC, with a steady increase of such a population over time.

Since 1995, our institution has overseen a blood culture surveillance program that identifies all patients with bloodstream infections. Data on epidemiology, clinical features, microbiology, treatment, and outcomes have been prospectively recorded. We analyzed all consecutive episodes of BSI occurring in adults with SC between 1995 and 2019. For this study, we divided this 25-year period into five different five-year time spans. The ethics committee board of our institution approved this study.

### Definitions

Definitions for comorbidities, prognosis of underlying disease, and source of infections, have been previously provided (2, 4). The selection of antibiotic treatment for the patients was conducted by the attending physicians without any influence from the article’s investigators. Treatment protocols take into account a syndromic antibiotic approach (such as pneumonia, urinary tract infection, etc.), the origin of the infection (community-acquired or nosocomial), and the local ecology. For those patients with neutropenia, the protocols align with national guidelines (13).

Neutropenia was defined as an absolute neutrophil count ≤500 cells/mm. Appropriate empiric therapy was considered when the patient received at least one *in vitro* active empiric antimicrobial agent, and the dosage and route of administration were in accordance with current medical standards. Mortality was defined as death occurring within 30 days of bacteremia onset. Death was considered related to the BSI if it occurred before the resolution of symptoms or signs, or within 7 days of the onset of bacteremia, and there was no other explanation. The following Gram-negative bacilli (GNB) were classified as MDR: (1) extended-spectrum beta-lactamase (ESBL)-producing Enterobacterales; (2) Carbapenem-resistant Enterobacterales; and (3) non-fermenting GNB that are resistant to at least three classes of these antibiotics, namely carbapenems, ureidopenicillins, cephalosporins (ceftazidime and cefepime), monobactams, aminoglycosides, and fluoroquinolones. Extensively drug-resistant (XDR)-GNB were those non-susceptible to ≥1 agent in all but ≤2 categories (14). The following Gram-positive cocci (GPC) were classified as MDR: (1) methicillin-resistant *Staphylococcus aureus* (MRSA) and (2) vancomycin-resistant enterococci (VRE).

### Microbiologic methods

Blood samples were processed using the BACTEC 9240 system or BACTEC FX system (Becton Dickinson Microbiology Systems), with a 5-day incubation period. Isolates were identified by standard techniques. A single positive culture was considered as significant if the microbe was clinically relevant or isolated in blood cultures. Of note, for BSI, due to coagulase-negative staphylococci (CoNS) whereby no catheter tip culture was available, at least two sets of positive blood cultures from different venipuncture sites were required to be considered as significant. Antimicrobial susceptibility testing was performed with either an automated microdilution system (MicroScan WalkAway, Beckman Coulter, West Sacramento, CA, USA; Phoenix system, Becton Dickinson, Franklin Lakes, NJ, USA), a semiautomated method (Sensititre, Thermo Fisher Scientific), or gradient strips (Etest; AB BIODISK, Solna, Sweden/bioMérieux, Marcy-l’Étoile, France). ESBLs were suspected by minimum inhibitory concentration (MIC) results and confirmed by double-disc synergy testing (15). Carbapenemase-producing Enterobacterales were phenotypically detected by the modified carbapenem inactivation method (mCIM) (16), in combination with the NG-Test CARBA 5 lateral flow immunoassay (NG Biotech, France) to detect the five most prevalent carbapenemases [*Klebsiella pneumoniae* carbapenemase (KPC), OXA-48-like, Verona integron-encoded metallo-β-lactamase (VIM), imipenemase (IMP), and New Delhi metallo-β-lactamase (NDM)] (17). We used the current Clinical and Laboratory Standards Institute (CLSI) (from 1995 to 2009) and European Committee on Antimicrobial Susceptibility Testing (EUCAST) breakpoints (from 2010 to 2019) for each year to define the susceptibility or resistance to these antimicrobial agents; intermediate susceptibility was considered as resistance.

### Statistical analysis

Categorical variables were detailed as counts and percentages, whereas continuous variables were described as either means and standard deviations (SDs) or medians and interquartile ranges (IQRs). The Pearson’s χ^2^ test and either the Mann-Whitney U test or the Student’s *t*-test according to the variable distribution were employed to compare the distribution of categorical and continuous variables, respectively. The Ordinary Least Squares (OLS) linear regression model was applied to analyze the temporal trend. We calculated the annual percentage change (APC) and its 95% confidence intervals (95% CI). A trend was identified as increasing with a positive regression coefficient or decreasing with a negative one when *P* < 0.05. Additionally, diagnostic tests were conducted to assess the assumptions for autocorrelation and the validity of the linear regression model. Chi-square for trends was used to compare the different time spans. A multivariate regression model (step-forward procedure) was used to identify independent risk factors for MDR BSI. We constructed a regression model with the inclusion of the MDR BSIs as the dependent variable. For the independent variables, we chose those parameters that showed predictive value using univariate analysis (*P* < 0.05). The goodness-of-fit of the multivariate model was assessed with the Hosmer-Lemeshow test and the area under the receiver operating characteristic curve. Our research utilized IBM SPSS Statistics for Windows (Version 25.0, Armonk, NY: IBM Corp.) and the Python programming language for data analysis.

## RESULTS

### Cohort and BSI characteristics

We documented a total of 5,896 patients with SC and BSI. The median age of the patients was 68 (IQR 59–76) years, and 3,835 (65.0%) were males. The most common SC was colorectal cancer (19.5%), followed by hepatobiliary cancers (14.3%), and urothelial cancer (13.5%). Only 333 (5.7%) patients were neutropenic and 947 (16.5%) had previously received corticosteroids. The urinary source of BSI was the most common (23.2%), followed by catheter-related BSI (20.8%) and biliary-tract BSI (10.9%). Table 1 shows the changes in patients and BSI characteristic over the study period.

**TABLE 1:** Main characteristics of patients, source of BSI and outcomes over the five-year time spans^*b*^^,^^*c*^

Number of patients with SC^*a*^ (%)	1995–1999 874 (14.8)	2000–2004 1147 (19.5)	2005–2009 1302 (22.1)	2010–2014 1254 (21.3)	2015–2019 1319 (22.4)	Overall 5896 (100)	*P*-value for trend
Male sex	524 (60.0)	730 (63.6)	852 (65.4)	853 (68.0)	876 (66.4)	3835 (65.0)	<0.001
Median (IQR) age, in years	68 (58–76)	68 (58–77)	68 (52–84)	68 (60–76)	68 (59–77)	68 (60–77)	NS
Baseline disease							
Diabetes mellitus	137 (15.7)	177 (15.4)	194 (14.9)	249 (19.9)	247 (18.7)	1004 (17.0)	0.002
Chronic hepatopathy	106 (12.1)	146 (12.7)	116 (8.9)	131 (10.4)	97 (7.4)	596 (10.1)	<0.001
Chronic heart disease	58 (6.6)	104 (9.1)	109 (8.4)	138 (11.0)	123 (9.3)	532 (9.0)	0.014
Chronic lung disease	65 (7.4)	78 (6.8)	85 (6.5)	112 (8.9)	91 (6.9)	431 (7.3)	NS
Chronic kidney disease	47 (5.4)	31 (2.7)	67 (5.1)	82 (6.5)	124 (9.4)	351 (6.0)	<0.001
Type of SC^*a*^							
Colorectal cancer	121 (13.8)	216 (18.8)	294 (22.6)	284 (22.6)	235 (17.8)	1150 (19.5)	0.015
Hepatobiliary cancers	134 (15.3)	166 (14.5)	169 (13)	175 (14)	198 (15)	842 (14.3)	NS
Urothelial cancer	83 (9.5)	125 (10.9)	195 (15.0)	184 (14.7)	210 (15.9)	797 (13.5)	<0.001
Pancreatic cancer	62 (7.1)	65 (5.7)	99 (7.6)	119 (9.5)	150 (11.4)	495 (8.4)	<0.001
Breast cancer	91 (10.4)	92 (8.0)	100 (7.7)	73 (5.8)	91 (6.9)	447 (7.6)	0.002
Lung cancer	61 (7.0)	106 (9.2)	95 (7.3)	83 (6.6)	95 (7.2)	440 (7.5)	NS
Prostate cancer	48 (5.5)	90 (7.8)	90 (6.9)	94 (7.5)	106 (8.0)	428 (7.3)	NS
Gastro-oesophageal cancer	82 (9.4)	51 (4.4)	84 (6.5)	61 (4.9)	70 (5.3)	348 (5.9)	0.003
Gynecological cancer	43 (4.9)	62 (5.4)	65 (5)	59 (4.7)	84 (6.4)	313 (5.3)	NS
Other^*a*^	185 (21.2)	222 (19.4)	151 (11.6)	163 (13)	192 (14.6)	913 (15.5)	<0.001
Clinical conditions							
Neutropenia	78 (9)	64 (5.6)	59 (4.6)	66 (5.4)	66 (5.1)	333 (5.7)	0.002
Prior corticosteroids within the last 3 months.	141 (16.2)	237 (20.8)	154 (12.6)	201 (16.9)	214 (16.5)	947 (16.5)	NS
Central venous catheter	339 (78.8)	457 (58.1)	599 (55.1)	515 (76.8)	512 (74.9)	2422 (66.2)	<0.001
Prior antibiotic therapy (1 month)	319 (36.5)	372 (32.4)	488 (37.5)	565 (45.1)	645 (48.9)	2389 (40.5)	<0.001
Antibiotic therapy at BSI onset	288 (33)	276 (24.1)	308 (23.7)	344 (27.4)	315 (23.9)	1531 (26)	0.003
Most frequent source of BSI							
Urinary	146 (16.7)	240 (20.9)	304 (23.3)	317 (25.3)	362 (27.4)	1369 (23.2)	<0.001
Catheter	211 (24.1)	218 (19.0)	309 (23.7)	287 (22.9)	200 (15.2)	1225 (20.8)	<0.001
Endogenous/Unknown	176 (20.1)	270 (23.5)	281 (21.6)	207 (16.5)	231 (17.5)	1165 (19.8)	<0.001
Biliary tract	87 (10.0)	104 (9.1)	135 (10.4)	127 (10.1)	187 (14.2)	640 (10.9)	<0.001
Intra-abdominal	71 (8.1)	99 (8.6)	84 (6.5)	97 (7.7)	113 (8.6)	464 (7.9)	NS
Pulmonary source	77 (8.8)	90 (7.8)	71 (5.5)	55 (4.4)	64 (4.9)	357 (6.1)	<0.001
Septic shock	119 (13.7)	189 (16.5)	166 (12.8)	191 (15.3)	195 (15.0)	860 (14.7)	NS

^*a*^ Including oropharyngeal (208), central nervous system (120), melanoma (111), sarcoma (84), and other solid cancers (289).

^*b*^ Abbreviations: SC, solid cancer; BSI, bloodstream infection.

^*c*^ All percentages are according to the number of patients with SC in the defined period.

### Causative microorganisms and antibiotic resistance

A total of 6,117 different pathogens were isolated in the 5,896 patients with SC and BSI. Overall, 36.3% of BSI were due to GPC and 60.4%, to Gram-negative bacilli (GNB). The most common GNB were *Escherichia coli* (1,637, 26.8%), *Klebsiella* spp. (760, 12.4%), and *Pseudomonas aeruginosa* (525, 8.6%). Coagulase-negative staphylococci (CoNS) (701, 11.5%), *Enterococcus* spp. (584, 9.5%), *S. aureus* (470, 7.7%), and *Streptococcus* spp. (464, 7.6%) were the most frequent GPC. Additionally, there were 203 (3.3%) episodes of candidemia and 617 (10.1%) polymicrobial episodes. Table 2 details causative microorganisms and MDR isolates over time.

**TABLE 2:** Changes over time in BSI epidemiology in patients with solid cancer^*e*^

Number of BSI (%) per period	1995–1999 *n* = 900 (14.7)	2000–2004 *n* = 1183 (19.3)	2005–2009 *n* = 1352 (22.1)	2010–2014 *n* = 1325 (21.7)	2015–2019 *n* = 1357 (22.2)	Overall *n =* 6117 (100)	*P*-value for trend
Gram-positive cocci	470/900 (52.2)	503/1183 (42.5)	463/1352 (34.2)	394/1325 (29.8)	389/1357 (28.7)	2219/6117 (36.3)	<0.001
Coagulase-negative staphylococci	184/900 (20.4)	193/1183 (16.3)	151/1352 (11.2)	89/1325 (6.7)	84/1357 (6.2)	701/6117 (11.5)	<0.001
*Enterococcus* spp.	83/900 (9.2)	81/1183 (6.8)	123/1325 (9.1)	149/1325 (11.2)	148/1357 (10.9)	584/6117 (9.5)	0.002
*E. faecali*/Enterococcus spp.	66/83 (79.5)	61/81 (75.3)	88/123 (71.5)	89/149 (59.7)	68/148 (45.9)	372/584 (63.7)	NS
*E. faecium/Enterococcus* spp*.*	12/83 (14.4)	14/81 (17.3)	34/123 (27.6)	55/149 (36.9)	78/148 (49.4)	193/584 (33.0)	<0.001
Vancomycin-resistant *Enterococcus*/*Enterococcus* spp.	2/83 (2.4)	3/81 (3.7)	3/123 (2.4)	5/149 (3.4)	2/148 (1.4)	15/584 (2.6)	NS
*S. aureus*	95/900 (10.6)	119/1183 (10.1)	103/1352 (7.6)	78/1325 (5.9)	75/1357 (5.5)	470/6117 (7.7)	<0.001
MRSA^*a*^/*S. aureus*	27/95 (28.4)	24/119 (20.2)	24/103 (23.3)	12/78 (15.4)	13/75 (17.3)	100/470 (21.3)	<0.001
*Streptococcus* spp.	108/900 (12.0)	110/1183 (9.3)	86/1352 (6.4)	78/1325 (5.9)	82/1357 (6.0)	464/6117 (7.6)	<0.001
Gram-negative bacilli (GNB)	400/900 (44.4)	653/1183 (55.2)	833/1352 (61.6)	878/1325 (66.3)	931/1357 (68.6)	3695/6117 (60.4)	<0.001
*E. coli*	209/900 (23.2)	326/1183 (27.6)	355/1352 (26.3)	367/1325 (27.7)	380/1357 (28.0)	1637/6117 (26.8)	0.024
ESBL^*a*^ *E. coli*/*E. coli*	4/209 (1.9)	22/326 (6.7)	57/355 (16.1)	71/367 (19.3)	79/380 (20.8)	233/1637 (14.2)	<0.001
Carbapenem-resistant *E. coli*/*E. coli*	0	0	3/355 (0.8)	1/367 (0.3)	5/380 (1.3)	9/1637 (0.5)	0.021
*Klebsiella* spp.	46/900 (5.1)	70/1183 (5.9)	180/1352 (13.3)	198/1325 (14.9)	266/1357 (19.6)	760/6117 (12.4)	<0.001
ESBL^*a*^ *Klebsiella* spp/*Klebsiella* spp	1/46 (2.2)	7/70 (10)	38/180 (21.1)	50/198 (25.3)	95/266 (35.7)	191/760 (25.1)	<0.001
Carbapenem-resistant *Klebsiella* spp/*Klebsiella* spp.	0	0	1/180 (0.6)	11/198 (5.6)	24/266 (9.0)	36/760 (4.7)	<0.001
*P. aeruginosa*	51/900 (5.7)	100/1183 (8.5)	119/1352 (8.8)	142/1325 (10.7)	113/1357 (8.3)	525/6117 (8.6)	0.010
MDR *P. aeruginosa^b^*/*P. aeruginosa*	6/51 (11.8)	8/100 (8.0)	24/119 (20.2)	25/142 (17.6)	11/113 (9.7)	74/525 (14.1)	NS
XDR *P. aeruginosa*^*c*^/*P. aeruginosa*	4/51 (7.8)	4/100 (4.0)	19/119 (16.0)	24/142 (16.9)	11/113 (9.7)	62/525 (11.8)	0.028
*Enterobacter* spp.	19/900 (2.1)	42/1183 (3.6)	75/1352 (5.5)	70/1325 (5.3)	62/1357 (4.6)	268/6117 (4.4)	0.002
ESBL^*a*^ *Enterobacter* spp./*Enterobacter* spp.	5/19 (26.3)	8/42 (19.0)	29/75 (38.7)	16/70 (22.9)	23/62 (37.1)	81/268 (30.2)	0.013
Other Enterobacterales	42/900 (4.7)	74/1183 (6.3)	63/1352 (4.7)	64/1325 (4.8)	71/1357 (5.2)	314/6117 (5.1)	NS
ESBL^*a*^ producing other Enterobacterales/other Enterobacterales	5/42 (11.9)	8/74 (10.8)	5/63 (7.9)	8/64 (12.5)	9/71 (12.7)	35/314 (11.1)	NS
Carbapenem-resistant *Klebsiella* spp./*Klebsiella* spp.	0	0	1/180 (0.6)	11/198 (5.6)	24/266 (9.0)	36/760 (4.7)	<0.001
*S. maltophilia*	7/900 (0.8)	8/1183 (0.7)	7/1352 (0.5)	9/1325 (0.7)	16/1357 (1.2)	47/6117 (0.8)	NS
Polymicrobial^*d*^	93/900 (10.3)	113/1183 (9.6)	109/1352 (8.1)	160/1325 (12.1)	142/1357 (10.5)	617/6117 (10.1)	NS
Candidemia	30/900 (3.3)	27/1183 (2.3)	56/1352 (4.1)	53/1325 (4.0)	37/1357 (2.7)	203/6117 (3.3)	NS
MDR-GPC^*a*^	29/470 (6.2)	27/503 (5.4)	27/463 (5.8)	17/394 (4.3)	15/389 (3.9)	115/2219 (5.2)	<0.001
MDR-GNB^*a*^	29/400 (7.3)	61/653 (9.3)	157/833 (18.8)	172/878 (19.6)	225/931 (24.2)	644/3695 (17.4)	<0.001
Any MDR^*a*^	58/900 (6.4)	88/1183 (7.4)	184/1352 (13.6)	189/1325 (14.3)	240/1357 (17.7)	759/6117 (12.4)	<0.001

^*a*^ Abbreviations: MRSA, methicillin-resistant *Staphylococcus aureus;* ESBL, extended-spectrum beta-lactamase; MDR, multiple drug-resistant; XDR, extensively drug-resistant; GPC, Gram-positive cocci; GNB, Gram-negative bacilli.

^*b*^ MDR *P. aeruginosa* is defined as non-susceptible to at least one agent in three or more antimicrobial categories.

^*c*^ XDR *P. aeruginosa* is defined as non-susceptibility to at least one agent in all but two or fewer antimicrobial categories.

^*d*^ Polimicrobial infections: 263 were GPC and GNB infections, 69 were caused by two or more GPC, four were polymicrobial GPC (including at least two GPC), and at least one GNB at the same time, 12 episodes were caused by candidemia and GPC, 15 episodes were caused by candidemia and GNB, and four episodes of candidemia included at least two different species of Candida, there were also 250 episodes of polymicrobial GNB infections.

^*e*^ Antimicrobial categories and agents used to define MDR and XDR *P. aeruginosa* include aminoglycosides, antipseudomonal carbapenems, antipseudomonal cephalosporins, antipseudomonal fluoroquinolones, antipseudomonal penicillins + β-lactamase inhibitors, monobactams, phosphonic acids, and polymyxins.

**Fig 1:**
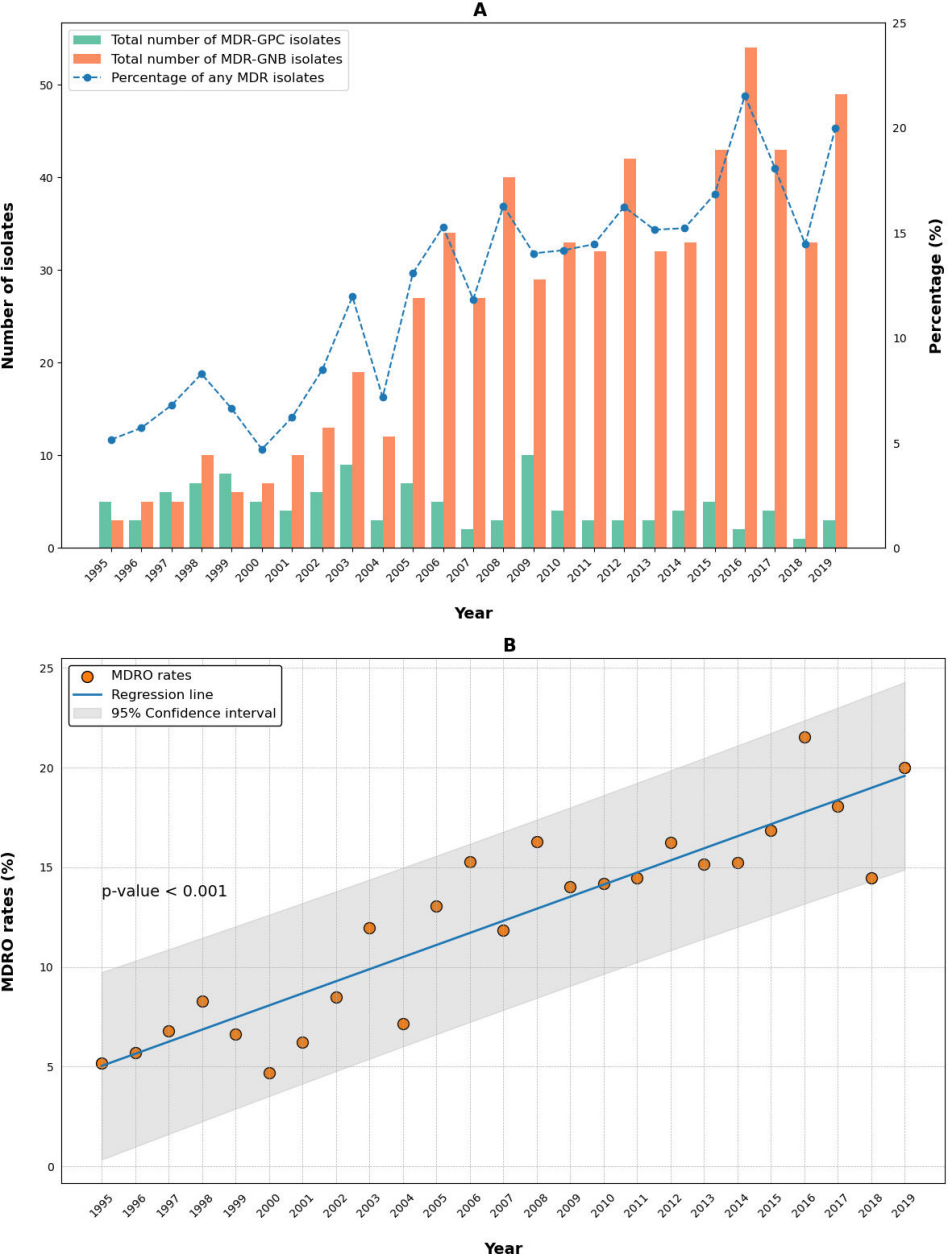
Temporal trends in annual incidence rates of MDRO in bloodstream infections over 25 years. MDRO, multidrug-resistant organism; MDR-GPC, multiple drug-resistant Gram-positive cocci; MDR-GNB, multiple drug-resistant Gram-negative bacilli.

Over the study period, there were 759 (12.4%) BSI episodes caused by MDR isolates, 115 (2.0%) by MDR-GPC, and 644 (10.9%) by MDR-GNB. A significant increase in the rates of multidrug-resistant organisms (MDROs) was observed over the study period (*P* < 0.001), with an APC of 0.57% (95% CI 0.49; 0.69) (Fig. 1). MDR-GPC significantly decreased over time (*P* < 0.001) due to a decline in the prevalence of BSI caused by MRSA (*P* < 0.001). Conversely, BSI caused by MDR-GNB increased (*P* < 0.001), mainly secondary to the rise in ESBL-producing *Klebsiella* spp. and ESBL-producing *E. coli* (*P* < 0.001 for both).

**Fig 2:**
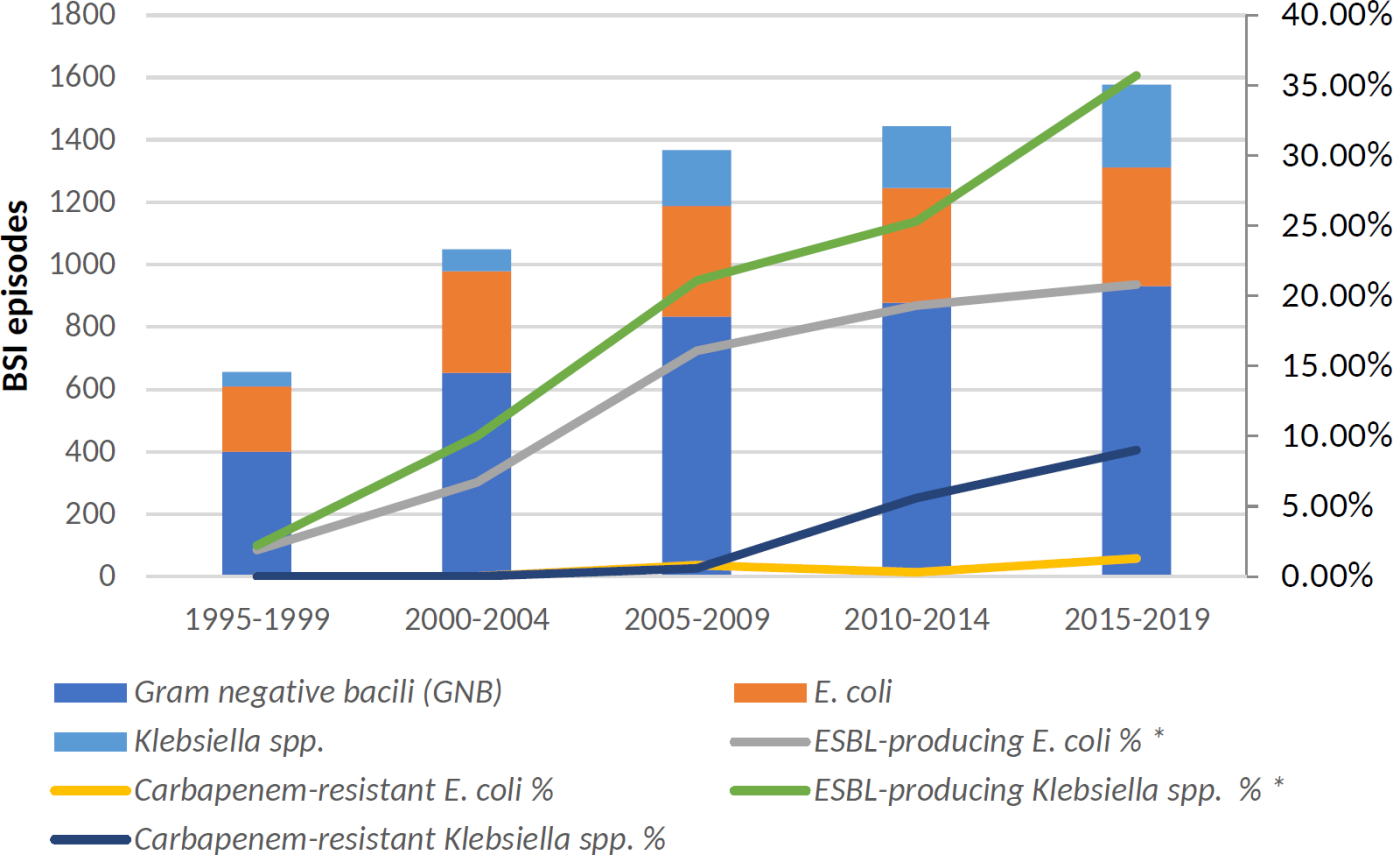
Changes over time in *E. coli* and *Klebsiella* spp. resistance (including ESBL-producing and carbapenem-resistant isolates). ESBL, extended-spectrum beta-lactamase; MDR, multidrug-resistant; XDR, extensively drug-resistant; and BSI, bloodstream infection.

**Fig 3:**
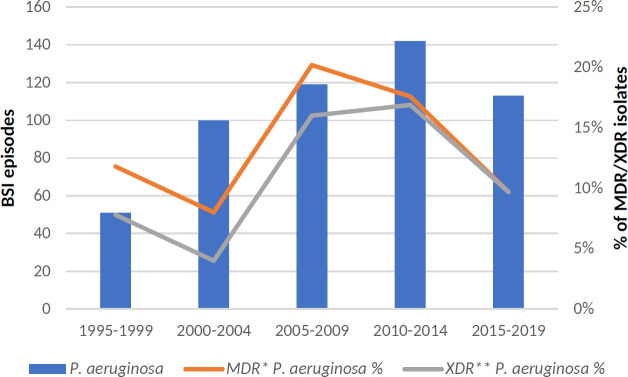
Changes over time in *P. aeruginosa* resistance to the main antipseudomonal agents. MDR, multidrug-resistant; XDR, extensively drug-resistant; and BSI, bloodstream infection.

Of the 644 MDR-GNB BSI documented, the most common isolates were 233 (3.8% of total BSIs) ESBL-producing *E. coli*, 191 (3.1%) ESBL-producing *Klebsiella* spp., and 74 (1.2%) MDR *P. aeruginosa*. Fig. 2 and 3 detail the changes in BSI antimicrobial resistance caused by the main Enterobacterales and *P. aeruginosa* over time, respectively.

In the last time span of years (2015–2019), 20.8% of *E. coli* isolates and 35.7% of *Klebsiella* spp. were ESBL producers and 1.3% and 9.0%, respectively, were carbapenem-resistant. Regarding *P. aeruginosa*, 21.2% of all isolates were resistant to one of the most common antipseudomonal beta-lactams (ceftazidime, piperacillin-tazobactam and/or meropenem); 14.1% were MDR strains and 11.8% met criteria for XDR.

### Appropriateness of empiric antibiotic therapy and mortality

Over the study period, 1,520 (24.8%) BSI episodes received IEAT. In Enterobacterales, IEAT was 15.2% and more frequent among ESBL (42% vs 15.2%, *P* < 0.001) and carbapenem-resistant isolates (51.1% vs 15.2%, *P* < 0.001). In *P. aeruginosa*, 31.3% episodes received IEAT, and it was significantly more frequent in MDR (58.1% vs 31.4%, *P* < 0.001) and XDR (62.9% vs 31.4%) *P. aeruginosa*. Table 3 summarises the changes over time on IEAT rates.

**TABLE 3:** Treatment and outcomes^*e*^

Number of BSI (%) per period	1995–1999 *n* = 900 (14.7)	2000–2004 *n* = 1183 (19.3)	2005–2009 *n* = 1352 (22.1)	2010–2014 *n* = 1325 (21.7)	2015–2019 *n* = 1357 (22.2)	Overall *n* = 6117 (100)	*P*-value for trend
IEAT	234/900 (26.0)	288/1183 (24.3)	358/1352 (26.5)	355/1325 (26.8)	285/1319 (21.0)	1520/6117 (24.8)	NS
IEAT Enterobacterales / Enterobacterales BSI	27/316 (8.5)	68/512 (13.3)	119/673 (17.7)	131/699 (18.7)	107/779 (13.7)	452/2979 (15.2)	<0.001
IEAT ESBL / ESBL BSI	5/15 (33.3)	15/45 (33.3)	63/129 (48.8)	78/145 (53.8)	66/206 (32.0)	227/540 (42.0)	<0.001
IEAT carbapenem-resistant/ carbapenem-resistant BSI	0 (0.0)	0 (0.0)	3/4 (75.0)	5/12 (41.7)	15/29 (51.7)	23/45 (51.1)	<0.001
IEAT *P. aeruginosa* / *P. aeruginosa*	9/51 (17.6)	31/100 (31.0)	42/119 (35.3)	49/142 (34.5)	34/113 (30.1)	165/525 (31.4)	0.02
IEAT MDR *P. aeruginosa^b^*^,*c*^/ MDR *P. aeruginosa^b^^,^^c^*	2/6 (33.3)	5/8 (62.5)	14/24 (58.3)	14/25 (56.0)	8/11 (72.7)	43/74 (58.1)	NS
IEAT XDR *P. aeruginosa^b^*^,^^*c*^/ / XDR *P. aeruginosa^b^^,^^c^*	2/4 (50.0)	3/4 (75.0)	12/19 (63.2)	14/24 (58.3)	8/11 (72.7)	39/62 (62.9)	NS

Number of patients with SC^a^ (%)^d^	1995–1999 869 (15.0)	2000–2004 1,139 (19.7)	2005–2009 1,284 (22.2)	2010–2014 1,238 (21.4)	2015–2019 1,257 (21.7)	Overall 5,787 (100)	*P*-value for trend
Related mortality	133 (15.3)	126 (11.1)	128 (10)	134 (10.8)	136 (10.8)	657 (11.4)	0.009
30-day mortality	157 (18.1)	158 (13.9)	198 (15.4)	210 (17.0)	220 (17.5)	943 (16.3)	NS
30-day mortality Enterobacterales / 30-day mortality	34/157 (21.7)	51/158 (32.3)	68/198 (34.3)	68/210 (32.4)	78/220 (35.5)	299/943 31.7)	NS
30-day mortality *P. aeruginosa* / 30-day mortality	9/157 (5.7)	21/158 (13.3)	17/198 (8.6)	17/210 (8.1)	12/220 (5.5)	76/943 (8.1)	NS

^*a*^ Abbreviations. MRSA, methicillin-resistant *Staphylococcus aureus;* ESBL, extended-spectrum beta-lactamase; MDR, multidrug-resistant; XDR, extensively drug-resistant; GPC, Gram-positive cocci; GNB, Gram-negative bacilli.

^*b*^ MDR *P. aeruginosa* is defined as non-susceptibility to at least one agent in three or more antimicrobial categories.

^*c*^ XDR *P. aeruginosa*/XDR is defined as non-susceptible to at least one agent in all but two or fewer antimicrobial categories.

^*d*^ Some patients’ data were not included due to loss in follow-up (some patients might had been transferred to another hospital or facility).

^*e*^ Antimicrobial categories and agents used to define MDR and XDR *P. aeruginosa* include aminoglycosides, antipseudomonal carbapenems, antipseudomonal cephalosporins, antipseudomonal fluoroquinolones, antipseudomonal penicillins + β-lactamase inhibitors, monobactams, phosphonic acids, and polymyxins.

Thirty-day mortality was 16.3% and remained stable throughout the study period (*P* = 0.355). Mortality was significantly higher in patients receiving IEAT (22.9% vs 14%, *P* < 0.001).

### Risk factors for BSI caused by MDR strains

In the multivariate analysis, the independent risk factors for a MDR BSI in our cohort of patients were: prior antibiotic use (OR 2.93, CI 2.34–3.67), BSI occurring during antibiotic treatment (OR 1.46, CI 1.18–1.81), biliary source of BSI (OR 1.84, CI 1.34–2.52), urinary source of BSI (OR 1.86, CI 1.43–2.43), and period of admission (OR 1.28, CI 1.18–1.38). Community-acquired infection was related with lower risk of MDR BSI (OR 0.57, CI 0.39–0.82). The goodness of fit of the multivariate model was assessed by the Hosmer-Lemeshow test (0.644), and the discriminatory power of the score, as evaluated by the area under the receiver operating characteristic curve, was 0.72 (95% CI, 0.70–0.74), demonstrating a good ability to predict MDR BSI.

## DISCUSSION

This article shows that several changes in BSI epidemiology have occurred in patients with SC over the past decades. These differences represent a current challenge in terms of empiric antibiotic treatment of this population, as made evident by the high percentage of patients with GNB infections who receive IEAT, and elevated 30-day mortality.

The documented changes in BSI epidemiology may be due to various reasons. There are host factors that have changed over the years: in historical studies (2, 6), breast, hepatobiliary, and lung cancer were the most common types of cancer in patients who developed BSI. Here, the most frequent location of SC was colorectal, followed by hepatobiliary and urothelial. Infections often depend on the localization and size of the SC, or the surgeries or medical devices required for its management (18). Important variations in bacteremia sources have also occurred (19). It is worth highlighting the decrease in BSI of pulmonary origin and the increase in infections of urinary origin, in line with the changes in the prevalence of cancer locations. Catheter sepsis is becoming less frequent (20), perhaps due to the better management of indwelling catheters (21). All these changes make it easy to understand the decline in CGP infections, except for *Enterococcus faecium* infection, and the rise of GNB infections, particularly of *Klebsiella* spp.

The increase in *E. faecium* infections has been described previously (22). The possible causes of such emergence have been hypothesized as a result of the increasing use of beta-lactams and, in particular carbapenems due to the high prevalence of ESBL-Enterobacterales. Their low activity on its own against *Enterococci* favors its selection and expansion in the gut, increasing the risk of endogenous bacteremia (23).

Empiric coverage of GNB represents a major challenge in treating patients with SC. In our study, *E. coli* was the most frequently isolated pathogen, although there was a sharp increase for *Klebsiella* spp. The main concern associated with these infections has been the growing antimicrobial resistance trend over the years, as reported in other studies (8, 9). Specifically, there was an important rise in ESBL and, more recently, carbapenem-resistant isolates (24, 25). Additionally, the most common antipseudomonal antibiotics were frequently non-active, since more than half of the patients presenting MDR or XDR *P. aeruginosa* infection received inadequate antibiotic treatment (26).

Our study, in line with previous studies (6), documented that those patients receiving IEAT have higher mortality. Accordingly, it should be considered to rethink the empiric antibiotic regimen for these patients. Although there are some treatment guidelines for febrile neutropenia in oncologic patients (27, 28), most patients with cancer and BSI are not neutropenic. The results reported in this research justify the possible creation of a specific guideline to treat such an infection in patients with cancer, even if they do not present neutropenia in the near future. Our article provides data for improving risk assessment for MDR infections, those patients with prior antibiotic therapy, those having BSI while receiving antibiotics, as well as those with BSI from biliary or urinary sources that have a higher risk. Conversely, patients with community-acquired infections exhibit a lower prevalence of MDR BSI.

Specifically, recommendations concerning the best empiric approach for GNB infections should be reassessed, including the role of new antibiotics and/or combination treatments with empiric antibiotics. Considering that the universal use of new antibiotics is not affordable, clinicians should explore innovative ways of identifying patients with a high risk of MDR-GNB BSI or prescribing broad-spectrum antibiotics followed by early de-escalation strategies, with the primary goal of reducing the inappropriate use of antibiotics in this population, alongside establishing a policy aimed at rationalizing antibiotic usage.

Our study strengths arise from the fact that a senior specialist in infectious diseases collected and evaluated data from a 25-year time span. However, our study has some limitations. It is a single-centre study and data might differ from areas with other microbiologic ecology or varying SC prevalence. The lack of genetic assessment of the strains hindered a more precise description of the clonal changes over time. Our study did not focus on identifying factors associated with mortality, so despite describing an association between IEAT and mortality, other co-factors were not evaluated. Finally, MIC data were not used in our study to define MDROs.

In conclusion, the epidemiology in patients with SC and BSI has changed. An increase in MDR-GNB has compromised the current empiric antibiotic strategies used to date. The percentage of patients currently receiving inadequate empiric treatment is unacceptable, especially given that IEAT is associated with increased mortality. New treatment strategies such as using better tools to stratify patients by risk, prescribing empiric antibiotics with lower resistance rates, and initiating early de-escalation should be promoted.

## Supplementary Material

Reviewer comments

## SUPPLEMENTAL MATERIAL

OPEN PEER REVIEW reviewer-comments.pdf An accounting of the reviewer comments and feedback.

ASM does not own the copyrights to Supplemental Material that may be linked to, or accessed through, an article. The authors have granted ASM a non-exclusive, world-wide license to publish the Supplemental Material files. Please contact the corresponding author directly for reuse.
